# Two sides of breastfeeding support: experiences of women and midwives

**DOI:** 10.1186/1746-4358-5-20

**Published:** 2010-11-29

**Authors:** Caroline A Bäckström, Elisabeth I Hertfelt Wahn, Anette C Ekström

**Affiliations:** 1 School of Life Sciences, Skövde University, Skövde, Sweden

## Abstract

**Background:**

Midwives' support of breastfeeding in maternity wards has been proven to provide an impact on women's breastfeeding experiences. In previous studies women describe professional support unfavourably, with an emphasis on time pressures, lack of availability or guidance, promotion of unhelpful practices, and conflicting advice. Thus, the present study aims to investigate women's experiences and reflections of receiving breastfeeding support and midwives' experiences and reflections of giving breastfeeding support.

**Methods:**

This study was carried out in a county in southwestern Sweden during 2003-2004. A qualitative method, content analysis, was chosen for the study. The data came from interviews with women as well as interviews with midwives who were experienced in breastfeeding support.

**Results:**

The women's and midwives' experiences and reflections of receiving and giving breastfeeding support were conceptualized as one main theme: *"Individualized breastfeeding support increases confidence and satisfaction." *This theme contained three categories: "*The unique woman," "The sensitive confirming process*," and *"Consistency of ongoing support." *In order to feel confident in their new motherhood role, the women wanted more confirmation as unique individuals and as breastfeeding women; they wanted to be listened to; and they wanted more time, understanding, and follow-up from health professionals. In contrast, the midwives described themselves as encouraging and confirming of the women's needs.

**Conclusions:**

If health care professionals responded to the woman's unique needs, the woman felt that the breastfeeding support was good and was based on her as an individual, otherwise a feeling of uncertainty emerged. The midwives, however, expressed that they gave the women individual support, but they also expressed that the support came from different points of view, because the midwives interpreted women's signals differently.

## Background

Adopted by all WHO member states, the Global Strategy on Infant and Young Child Feeding provides a basis for protection, promotion, and support of breastfeeding, which is a public health priority [1]. Low rates and early cessation of breastfeeding have important adverse health and social implications for women, children, the community, and the environment. These factors also result in greater expenditure on national health care provision; and increased inequalities in health [2]. A Cochrane review of the associations between the support provided by health care professionals and the duration of breastfeeding showed that additional professional support was effective in prolonging *any breastfeeding*, but the effects of additional professional support on *exclusive breastfeeding *were less clear [3].

New mothers tend to think that physical availability of health care professionals will help them handle their anxieties regarding parenting and their changing role [4]. However, too little attention is paid to women's psychosocial problems, and health care professionals do not follow-up on the women's satisfaction with support [5]. Women describe professional support unfavourably, with an emphasis on time pressures, lack of availability or guidance, promotion of unhelpful practices, and conflicting advice [6]. According to earlier research in Sweden, women are dissatisfied with the breastfeeding information provided by health professionals [7]. In Sweden, women and their babies usually leave the hospital 6 to 72 hours after birth. In an earlier study in Sweden, it was shown that early discharge from hospital negatively influenced the duration of exclusive breastfeeding [8]. However, a Cochrane review shows that further research is needed in order to evaluate the influence of early discharge in relation to breastfeeding outcome [9]. The Global Strategy on Infant and Young Child Feeding points out the necessity for health care professionals to have evidence-based knowledge and skills in breastfeeding [1].

Previous studies have been performed in order to investigate whether a training intervention within the care team of antenatal and child health centres would improve maternal perception of support and strengthen maternal feelings for the baby [10-13]. The antenatal midwives and child health nurses were randomized (on a municipality level) to either the intervention (n = 36) or the control sites (n = 45). The intervention group was admitted to a process oriented training program. The training program was carried out from 1999-2000. The training program focused on breastfeeding experiences (both private and professional), attitudes within the group toward breastfeeding, and methods for organizing collaboration between prenatal centres and child health centres in order to establish continuity in breastfeeding support [10-13]. In order to evaluate the process-oriented training program, the women (n = 540) included in the previous studies had either been cared for by healthcare professionals at the intervention site as described above or by healthcare professionals at the control sites in 2000-2003. The women responded to questionnaires at three days, and three and nine months postpartum. The results showed that the process-oriented training program had a positive impact at the women's perception of professional support as well as on the women's feelings for and relationship with their babies. However, further research is needed to fully determine women's and midwives' views of breastfeeding support [3,13]. The aim of this study was to investigate women's experiences and reflections of receiving breastfeeding support, and midwives' experiences and reflections of giving breastfeeding support.

## Methods

A qualitative design with content analysis [14] was chosen for this study. It was decided that individual interviews were the most suitable data collection method, because the direct voices of the women and midwives can provide information about meaningful values and life experiences [15]. Content analysis is a stepwise process of categorization based on the expression of thoughts, feelings, and actions described throughout the text [14]. The intentions of the analytical process are to remain close to the words of the text and to bring out the contextual meanings. Content analysis can either be manifest or latent, depending on the depth and level of abstraction. Manifest content is about the actual text, while latent content describes what the text is talking about. For this study, latent content analysis was chosen to identify specific meanings in the women's and midwives' narratives about receiving and giving breastfeeding support. The stepwise process of categorization is presented under the heading "data generation and analysis".

### Study site and participants

This study was undertaken during 2003-2004 in a hospital maternity clinic in a south-western county of Sweden. The county consists of 13 municipalities and comprises urban, suburban, and rural districts, and contains 280,000 inhabitants. Approximately 2500 births occur annually at the maternity clinic. The prenatal and child health centres in the county serve both urban/suburban and rural districts. In Sweden, antenatal care is community-based. Midwives and the woman/couple will meet approximately 8 to 11 times during pregnancy; most women birth in hospital, and care in hospital is provided by midwives who are not known to the woman. The average length of hospital stay is between 6 hours and 4 days, and a child health nurse makes a home visit 7 to 10 days after the birth and maintains contact until the baby is old enough to start school at six years of age. This study is part of a larger intervention study that includes a process-oriented program on breastfeeding management and promotion for health professionals in prenatal and child health centres in the study area [10-13].

### Inclusion criteria for women

The women interviewed in this study were selected from participants in the earlier intervention study described previously. Quantitative designs were used for data collection in the previous studies [10-13]. The women, who were primiparas, were chosen through strategic sampling to obtain as varied data as possible in regard to social background, birth outcomes, time for discharge from hospital and different experiences of birth and support, in order to clarify women's views of breastfeeding support. Women who had given birth to babies with life-threatening diseases or malformations were excluded. The women were provided information about the study, its objectives, and the rights of the research participants in a leaflet and by telephone (by the third author AE).

### Inclusion criteria for midwives

A leaflet explaining the study, its objectives, and the rights of the research participants was sent to the hospital's maternity clinic. The head midwife of the maternity clinic was asked to contact midwives of varying ages and with a range of experience regarding breastfeeding support and health visits. Midwives were free to decline participation; four midwives voluntarily agreed to participate. The ages of the midwives who agreed to participate ranged from 35 to 49, and their experiences working with breastfeeding support ranged from 4 to 10 years. All midwives received written information about the study from the head midwife, and were provided with further details about the study prior to deciding to participate (by the first author CB).

### Interviews with the women

Interviews with the nine women were carried out between three and six months after birth and in the women's homes. The women were asked to describe their experiences of breastfeeding support. To put the women at ease and encourage them to share their experiences freely, the opening question was followed by such questions as [16]: "Please tell me more about. . .," "How do you mean . . . ?" and "Describe your feelings/thoughts when. . ." The interviews were audio-taped and lasted 45 to 60 minutes.

### Interviews with the midwives

Open-ended interviews with the midwives were conducted in a private room at the maternity ward. The interview began with a broad opening question: "What do you include in breastfeeding support?" Probing questions were then asked to clarify or deepen the answers. The interviews lasted 30 to 45 minutes, and were audio-taped [16].

### Analysis

The audio-taped interviews with the women and the midwives were transcribed verbatim and analyzed separately using latent content analysis [14]. The transcripts from the women and the midwives respectively were scrutinized several times, discussed, compared, and validated. Familiarity with the text was achieved by repeated reading. Words and sentences containing information relevant to the research questions were identified as meaning units, which were condensed and coded. The codes were grouped into subcategories and then categories. Data were further analyzed by reading across the categories, searching for new associations and meanings in the data. In the final step, findings were discussed and reflected upon, taking the research issues into account, and an overall theme emerged [14].

### Ethics approval

Ethics approval was received from the Regional Ethics Committee (protocol number: L188-99), and the clinical head of service for the hospital clinic provided access to undertake this study.

## Results

The women wanted to be seen as unique individuals by midwives and they wanted midwives to confirm their breastfeeding experiences. If midwives responded to the individual woman's needs, the woman felt positive about the breastfeeding support and that it was based on her as an individual. The women's and midwives' experiences and reflections of receiving and giving breastfeeding support were presented as a single main theme, *"Individualized breastfeeding support increases confidence and satisfaction." *This theme had three categories: "*The unique woman," *"*The sensitive confirming process*," and *"Consistency of ongoing support" *(Table 1). Each category and its subcategories were presented using direct quotations in a conversational format. A code number for each respondent is included after the quotation (Women: W1 to W9, Midwives: MW1 to MW4).

**Table 1:** Categories with subcategories and theme identified from interviews with women and midwives.

Categories and subcategories	Theme
**The unique woman**	
- Confirmation as a person and as a breastfeeding woman	
- Support to women, whether breastfeeding or not	
	
**The sensitive confirming process**	**Individualized breastfeeding support increases confidence and satisfaction**
- Observation	
- Confirmation, practical and physical support	
	
**Consistency of ongoing support**	
- Establish continuity	
- Follow-up	

### The unique woman

#### Confirmation as a person and as a breastfeeding woman

The *women *expressed a need for confirmation, because they were unsure about their breastfeeding competence and uncertain whether their breastfeeding was normal. The women expressing this issue mentioned that they felt safer when a health care professional was present during a breastfeeding session. Their self-confidence improved when the health care professionals confirmed a normal breastfeeding: *"I was wondering if he was nursing correctly and things like that but she was watching the entire time and said that everything was just like it should be . . ." *(W8)

The *midwives' *described that they adjust their breastfeeding support according to the needs of each unique woman. The primary source of information for the midwives was the woman herself. The participants said that they asked the woman about her view toward breastfeeding. The participants suggested that breastfeeding advice and support actually involve interplay between the midwife and the woman. The midwives also stressed the importance of taking time to listen to the wishes and needs of the individual woman in order to provide adequate breastfeeding support from the view of the woman: *" . . . in my initial talk with her, I ask her if she has any special wishes. In order to get an idea about the breastfeeding or her attitude, when she arrives here I ask her about it." *(MW4) The midwives stressed the importance of confirming the woman in other words, showing the woman respect both as a person and as a breastfeeding woman. The participants stated that women often lacked self-confidence and thus needed to be confirmed as breastfeeding women. The participants expressed different ways of confirming women. Some midwives confirm the woman verbally: *" . . . to support and verbally confirm that everything will be all right. That both breastfeeding and child seem adequate. That is the most important." *(MW1)

The *midwives *stressed the importance of asking women about any previous experiences of breastfeeding, because women with no previous breastfeeding experience need different support than those who have breastfed. The participants found that they themselves interpreted the signals of women differently than their colleagues. They stated that the solution to this issue was to listen to the woman and develop breastfeeding support according to the information provided: *"You may have received a report that the woman's breastfeeding was successful, but if you ask her she gives another version. I generally check on the status of the breastfeeding." *(MW1) *"This is very subtle, that is my interpretation of her words. Someone else might have heard something completely different . . . " *(MW2) One participant described how she developed an individual breastfeeding strategy. She explained in detail to the woman how to breastfeed, she recommended that they follow the individually adapted breastfeeding program, and she informed her colleagues about the woman's process: *" . . . develop a breastfeeding strategy for each woman: just continue doing what we have been doing . . . and I informed the personnel out there as well." *(MW4) The participants referred women to care providers who could support them if future breastfeeding issues were to arise and stated: *"They must be encouraged that there is help, refer them to different locations, etc." *(MW1) In contrast, the women mentioned that health care professionals should follow up and monitor breastfeeding through recurring contact with the woman.

#### Support to women, whether breastfeeding or not

Some *women *felt that they were inadequate as women in cases when the health professionals did not confirm their ability to breastfeed. The same feeling of failure as a mother emerged when the health care professionals did not listen to or did not try to understand the woman's situation during breastfeeding. One woman felt that the health care professionals did not see her as an individual because of her inability to breastfeed; rather the health care professionals based their support on a woman's capability to breastfeed. According to the woman, health professionals should provide different styles of breastfeeding support to women who want to breastfeed but are incapable of it: *"They have tried to help me to the very last, but I still have not received the support where someone has spoken just the way I need, perhaps someone who understands in a different way how emotions become the way they are." *(W2) "*There is no breastfeeding support available. There is breastfeeding support but not for us who can't, who want to but can't. Breastfeeding support is only there for those who are able to and want to . . ." *(W1) "*I want to breastfeed but I am incapable of it. They just can't help me the way I would like them to . . . " *(W9).

The *midwives *stated that they provided individual support even to women who decided not to breastfeed, because they also needed support: *"To me breastfeeding support is support about breastfeeding irrespective of her choice . . . " *(MW1). To develop adequate individual breastfeeding support, the midwives observed a breastfeeding session, which is described in the following section.

### The sensitive confirming process

#### Observation

The *midwives *stated that it was important for them to observe a breastfeeding session in order to develop adequate individual breastfeeding support. They asked the women to call for them when it was time for breastfeeding: *" . . . and then she'll get in touch when the child starts signaling for food. I ask them if I may be present and if it's okay to watch. Now she has the child to her breast and I see right away how the child is laying on the breast, how she holds the child." *(MW4); *"I mention what we perhaps should change, amend, make better . . . " *(MW3) The majority of the participants said that encouragement, such as pointing out positive progress and enhancing the women's self-confidence, should be prominent in breastfeeding support. The midwives had different views on how to enhance the women's self-confidence. One midwife stated: *" . . . one provides support in a passive manner . . . " *(MW2), and took a step back in order for the woman to control the breastfeeding. Others were encouraging and praising: *"I support her. I basically cheer for her. To see how well she does . . . and always bring out everything positive in order to bring out the woman." *(MW2) One participant stated that the women only felt enhanced in their development when the women themselves contributed to the development. She encouraged the women to try breastfeeding and call for help only when necessary: *"Start yourself by laying the baby to your breast and try on your own. If it works out, it will enhance and provide better self-confidence." *(MW1)

The *women *focused more on *Confirmation, practical and physical support *instead of *observation*, as presented under the following section.

#### Confirmation, practical and physical support

*Women *strongly valued the health care professional's assistance during problems with breastfeeding. The women viewed adequate breastfeeding support as including information about breastfeeding timing, nursing techniques, or damaged nipples. They thought that practical breastfeeding support from the health care professionals should be in accordance with the unique needs of each woman: *"I rang and hoped they would come. I got help laying her down and they showed me how to hold her, how she should nurse and open her mouth and so forth, it was really good.*" (W5)

*Midwives *also considered practical advice important in adequate breastfeeding support. Practical advice, according to the midwives, includes information about timing of feeds, breastfeeding techniques, or management of nipple damage. The participants made a point of telling the women what they looked for during a breastfeeding session and why: *" . . . how the child is nursing, for example, why I want the child to nurse in a certain manner. What they should look for themselves, because they might have issues with damaged nipples, etc. What am I observing and why am I observing it ? " *(MW4); *" . . . I also show with my body what I mean." *(MW4); *" . . . provide some advice and suggestions about small changes that make a big difference. A lot of small observations you teach parents to make on own their own." *(MW3) Some participants confirm women by getting physically close: *" . . . I always sit down close, I do not stay far away, I almost sit on top of the woman. Or, if she sits on the edge of the bed, I will sit down on the edge of the bed. I have a lot of close physical contact with women. I hold them. I put my arm around them and show them with my body that I consider them adequate. I do not stand in the doorway and speak. I am very close . . . " *(MW1) Some also felt that they needed to show the woman how the child should lie by guiding its head to her breast: *" . . . sometimes you need to guide the head a little bit . . . my hand is sometimes able to guide it to the breast. (Pause) But at least it does not feel as if it is too rough . . . " *(MW1)

*Women *also raised the issue of physical contact and space, however, one woman was concerned about a midwife who demonstrated a breastfeeding technique in a careless manner and who she believed, upon reflection, did not help her breastfeed: *"Yes, at first I felt it was great when she helped me, but the more I thought about it I realized that I got no help at all, she tore at my breast, she pulled her head . . . but nothing was really gained, no, I really don't feel fully satisfied looking at it in retrospect." *(W6) The women experienced good practical support if the midwives showed adequate breastfeeding techniques and provided useful advice in a gentle way, but felt dissatisfied if the health professionals were heavy-handed.

### Consistency of ongoing support

#### Establish continuity

The *women *expressed that continuity is important in breastfeeding support. They thought that improved continuity by the health care professionals would establish confidence. Some women expressed insecurity as a result of contact with several different care providers during pregnancy, childbirth, and breastfeeding: *"Why is it not possible to meet the antenatal midwife at the maternity ward, the one you had established confidence in . . . when you meet a lot of new people it is difficult . . . " *(W3) They pointed to the fact that it took a long time to form a relationship with, and thus have confidence in one health care professional, and then have to start over again with someone else. The women expressed uncertainty when learning to breastfeed, and their uncertainty increased when different health care professionals provided different advice and information: *" . . . because one says one thing and another says something different, you get a little confused, that's how I feel." *(W7) The women thought that all health care professionals should use the same method of providing breastfeeding support. To experience adequate breastfeeding support, the women considered it important to have confidence in a health care professional before they openly expressed themselves to her: *"Why can't you have the same midwife as during pregnancy, the one you had confidence in instead of seeing a lot, yes, a lot of new faces you've never seen before ? " *(W5) Women's sense of insecurity decreases and their willingness to openly express themselves increases when a consensus about health professionals' views of support and practical methods is reached.

The *midwives *also stressed that it is very important in breastfeeding support to have consensus among the different views of care and practical methods used by health care professionals. To have continuity, the participants stated that it is important to base the support for women on the WHO guidelines regarding the ten steps to successful breastfeeding: *"How we perform work should be from a perspective that promotes breastfeeding. For instance, I feel that it corresponds to the ten steps. It feels as if we basically work according to those steps. And they are based on breastfeeding science . . . " *(MW4)

#### Follow-up

The importance of follow-up and continued breastfeeding support was reported by both women and midwives. Midwives stated that they provide follow-up for women and their breastfeeding, and they considered follow-up to be an important aspect of breastfeeding support. The midwives described follow-up as continually making contact with the woman, which enables observations of any changes in breastfeeding. The *midwives *are then able to support the woman by addressing problems and encouraging success based on these changes: *"At the next feed, I compare it with the previous and tell the woman about the latest development." *(MW4)

*Women *also talked about the importance of follow-up. They mentioned that health care professionals should monitor breastfeeding through recurring contact with the woman. One woman said: " . . . *they could at least ask once in a while . . . " *(W8)

### Individualized breastfeeding support increases confidence and satisfaction

The *women *wanted to be seen as unique individuals by the health professionals, and they also wanted the health care professionals to confirm their breastfeeding as normal or abnormal. If the health professionals responded to the woman's needs, the woman felt that the breastfeeding support was good and was based on her as an individual. If the woman experienced a lack of confirmation as a breastfeeding woman, and if she felt that she was not being supported as an individual, a feeling of uncertainty emerged. The midwives, however, expressed that they gave the women individual support, but they also expressed that the support came from different points of view, because each of the midwives interpreted women's signals differently.

## Discussion

Using content analysis, this study investigated women's and midwives' experiences and reflections of receiving and giving breastfeeding support. Individual interviews were chosen as the data collection method; interviews were able to catch the women's and midwives' narratives, which could provide information about meaningful values, experiences, and reflections [15]. Throughout the study, different steps were considered to enhance the trustworthiness of the study [17]. The study is limited by its small sample size, but the context and the participants are described as clearly as possible to facilitate the transferability of the results [15].

This study found differences in women's and midwives views of breastfeeding support. The women wanted more confirmation as unique individuals and as breastfeeding women. They wanted to be listened to and wanted more time, understanding, and follow-up from the health care professionals in order to feel confident in their new role as a breastfeeding woman. In contrast, the health care professionals described themselves as encouraging and confirming of the women's needs. According to the women, continuity in breastfeeding support is important in order for them to have confidence in the health professionals. When different health care professionals provided different advice, women described an increase in their uncertainty regarding breastfeeding. These results are in line with previous research that showed that if prenatal midwives and postnatal nurses participated in a process-oriented breastfeeding training program that included an intervention, the women they cared for were more satisfied with received emotional and informative support compared to the control group with traditional care, nine months postpartum [12].

In this study, the women stated that it was very important for them to be able to open up to the health care professionals in order to receive adequate breastfeeding support. Results from new research from Sweden show that women expressed a wish to be more involved and responsible than they actually were in consultations with the midwives [18]. At first, women wanted to be reassured by the midwife; to be able to take part in shaping the care-giving situation in interaction with the midwives [18]. However, if practical support is given by handling the mother's breast carelessly while simultaneously forcing her child's head to her nipple she looked back at the situation negatively. In addition, a child who is forced to the breast might refuse to breastfeed. This exemplifies two reasons for midwives to maintain a hands-off approach when giving breastfeeding support [19].

Previous research indicates that adequate breastfeeding support from health care professionals was not considered to be of the highest quality unless it was based on the woman's own needs and requirements. The ability of health care professionals to listen and understand will thus play an important role for women and their possibilities to breastfeed [5,20]. The Global Strategy on Infant and Young Child Feeding points out the necessity for health care professionals to have evidence-based knowledge and skills in breastfeeding [1]. The midwives stated that they supported the women whether or not they breastfed, while the women who did not breastfeed mentioned a feeling of not being seen, a lack of understanding, and a feeling of inadequacy as a woman. In order to gain insight into women's expectations regarding breastfeeding support, health care professionals must adopt a listening role [20]. If the midwife is able to remain with the woman when giving individual support, the midwife will see the effect of the action and thus be able to provide the breastfeeding support the woman asks for. According to earlier research, such support promotes the woman's personal values, feelings of self-confidence, and her confidence as mother in interaction with friends, relatives, and health care professionals. In order to satisfy these needs, health care professionals must intensify their focus on seeing the woman as a unique individual [21].

In order to strengthen women in their new role, it is important for health care professionals who provide breastfeeding support to reflect on their own attitudes [10], as well as to prioritize and to provide adequate evidence-based care to women. One strategy that has been shown to be effective in improving midwives' attitudes when providing breastfeeding support is a process-oriented breastfeeding approach using an empowerment approach [12,13,22]. An empowerment approach means that health care professionals provide women with the information, expertise, support, and skills they need to engage in interactive participation [23]. The empowerment approach [23] requires a shift from having power over the women to having power with the women. This is in line with WHO recommendations [1]. When women feel that midwives respect them and listen to them as individuals, they will feel confident about their health care. More research is needed to illuminate both the health care professionals' and the mothers' individual perspectives on empowerment in health care.

## Conclusion

If the health care professionals responded to the woman's unique needs, the woman felt that the breastfeeding support was good and was based on her as an individual otherwise a feeling of uncertainty emerged. The midwives, however, expressed that they gave the women individual support, but they also expressed that the support came from different points of views, because the midwives interpreted women's signals differently.

## Competing interests

The authors declare that they have no competing interests.

## Authors' contributions

CAB was active during the design, implementation (she held the interviews with the midwives), analysis, and writing. EIHW contributed during design, interpretation, and writing. ACE was active during the design, implementation (she held the interviews with the women), interpretation, and writing. All authors read and approved the final manuscript.
